# Predictive Role of MRI-Detected Modic Changes in the Chronicity and Severity of Low Back Pain: A Systematic Review and Meta-Analysis

**DOI:** 10.7759/cureus.107258

**Published:** 2026-04-17

**Authors:** Kirthika C. P., Senthil Kumar S., Venkata Sai P. M.

**Affiliations:** 1 Anatomy, Sri Ramachandra Institute of Higher Education and Research, Chennai, IND; 2 Radiology and Imaging Sciences, Sri Ramachandra Institute of Higher Education and Research, Chennai, IND

**Keywords:** disc degeneration, disc herniation, endplate changes, health care, inflammatory, low back pain, modic changes, mri images, spondylolisthesis, well-being

## Abstract

The main focus of this systematic review and meta-analysis was to establish a correlation between vertebral subchondral endplate changes (Modic changes, or MCs) and low back pain (LBP). This review also wanted to determine whether MCs serve as valid predictors of progressive degenerative spinal diseases and surgical outcomes. In accordance with PRISMA guidelines, an extensive literature search was conducted across major databases for peer-reviewed studies published up to 31 May 2025. The Joanna Briggs Institute (JBI) instrument and the Newcastle-Ottawa Scale (NOS) were used in assessing the quality of the studies. Data from 18 studies that involved about 4,637 patients were used in this review. The demographic risk factors, anatomical distribution, and clinical correlations were obtained from the included studies. In compliance with our primary objective, the meta-analysis revealed that increasing age is a notable risk factor, exhibiting a mean difference of 2.43 years (95% CI: 1.09 to 3.76) between the MC and non-MC groups. The majority of the males presented with MCs (OR: 1.51), although BMI did not exhibit a statistically significant link (p = 0.705). According to the secondary objective, MCs were common in the L4-L5 and L5-S1 intervertebral discs (IVDs). A significant correlation was identified between MCs and spondylolisthesis (OR: 50.50) as well as disc herniation. In terms of surgical outcomes, Modic type 3 changes (sclerotic) were linked to significantly lower fusion rates (54.5%) in posterior lumbar interbody fusion (PLIF) than in other types. It was known that smoking was a major risk factor for poor surgical recovery in people with type 1 MCs. MCs provide a "visible map" of the history of spinal degeneration, closely associated with mechanical instability and previous IV-disc herniations. Although MCs are a standalone indicator of chronic LBP, their subtypes have a considerable impact on the surgical outcomes. Preoperative MRI phenotyping, specifically distinguishing between inflammatory type 1 and sclerotic type 3, is essential for managing patient expectations and deciding on suitable surgical interventions to reduce risks such as cage subsidence or non-union.

## Introduction and background

Low back pain (LBP) hits millions every year. It disrupts well-being and drains healthcare resources [1]. This musculoskeletal spinal disorder is prevalent among the working population [2]. The components of the lumbar spine, such as soft tissue, vertebrae, and intervertebral discs (IVDs), are susceptible to various stresses that can cause LBP [3]. Research on Modic changes (MCs) has elucidated degenerative changes in the vertebral endplates and corresponding signal variations in MRI, demonstrating good specificity for discography in individuals with chronic low back pain (CLBP) [4]. de Roos et al. studied the alterations in bone marrow signal intensity surrounding the endplates of vertebrae with degenerated discs [5]. Modic classified the changes in the vertebral endplate and bone marrow based on MRI imaging into three types. T1-weighted images (T1WIs) with hypointense signal and T2-weighted images (T2WIs) with hyperintense signal are seen in type 1 MCs, accompanied by endplate inflammation indicative of oedema and increased vascularity. Type 2 exhibits hyperintense signals in T1WI and T2WI, signifying the substitution of red bone marrow with adipose tissue within the vertebral body. Type 3 often denotes the ultimate phase of vertebral endplate alterations, characterized by hypointense signals in T1WI and T2WI, resulting from sclerotic modifications in the endplate of the adjacent vertebral body [6]. MCs are seen in the cases of spondylolisthesis, IV disc degeneration, and reduced IVD height [7]. Type 1 MCs are tightly linked to a previous disc herniation. Patients who had surgery for lumbar disc herniations had more changes in the subchondral endplate [8]. The changes were predictive of LBP. Lavanya et al. demonstrated that endplate changes are more commonly found at L4/L5 and L5/S1 in the lumbar spine. Modic types 1 and 3 were roughly equally prevalent, with type 2 being the most frequent. With advancing age, the changes were found at more levels, indicating the degenerative nature of the endplate [9]. The systematic review aims to correlate studies involving subchondral endplate changes (MCs) with LBP, assess the synergy between these two components, and evaluate the role of these changes in predicting the severity of LBP. The objective is to determine if the vertebral endplate changes could predict the progressive degenerative bone disorders.

## Review

Methodology

This systematic review adheres to the Preferred Reporting Items for Systematic Reviews and Meta-Analyses (PRISMA). Only published articles are included in this systematic review, necessitating no approval from the Internal Review Board (IRB).

Literature Search Strategies

**Figure 1:**
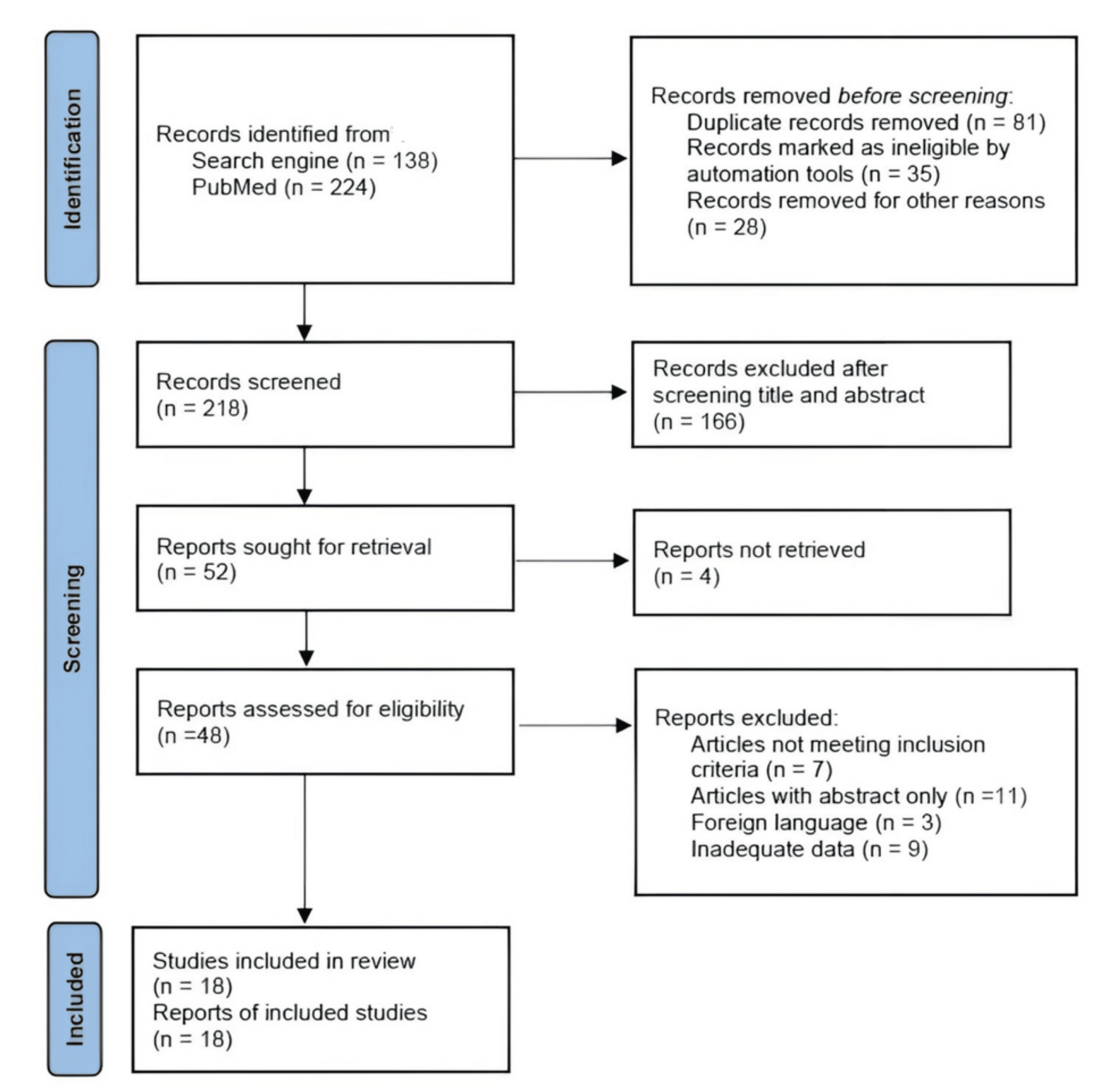
Preferred Reporting Items for Systematic Reviews and Meta-Analyses (PRISMA) diagram

A literature search for publications in peer-reviewed journals was carried out across the databases such as PubMed, Scopus, Medline, Embase, and Web of Science. The search terms were developed around the concepts of MCs and LBP, including combinations of the following keywords and subject terms: “Modic changes,” “endplate changes,” “low back pain,” “intervertebral disc,” and “degenerative disc disease.” Boolean operators (AND, OR) were used to combine search terms, e.g., (“Modic changes” OR “vertebral endplate changes” OR “endplate changes”) AND (“low back pain” OR “chronic low back pain”) AND (“intervertebral disc” OR “disc degeneration” OR “degenerative disc disease”). Careful screening of articles was done to ensure that only relevant articles were included in the study. Articles that were letters to the editor, book chapters, systematic reviews, and case reports were not included. Only full-text articles in English were included. Articles were searched for until 31 May 2025 and sorted (Figure 1).

Inclusion and Exclusion Criteria

Inclusion criteria: Studies on lumbar spine diseases and precursors to LBP, such as IVD herniation, spondylolisthesis, and degenerative bone disorders, were included. Studies on corrective surgical interventions done on the vertebra or the IVD were included. No limitations on age or gender were considered.

Exclusion criteria: Studies involving spine deformities, either congenital or acquired, were excluded. Studies involving patients with neurodevelopmental disorders or associated anomalies were not considered. Articles pertaining to the spine that included acquired infection, sarcoma, or chordoma were excluded.

Data Extraction and Quality Assessment

Two reviewers independently assessed every title and the abstract, and selected a full-text article for inclusion and exclusion. Disagreements were settled by consulting a third reviewer whenever needed.

The Newcastle-Ottawa Scale (NOS) was used to evaluate three key aspects: selection of study groups, comparability of those groups, and assessment of the outcome or exposure of interest. Those studies with more stars indicated better methodological quality. The Joanna Briggs Institute (JBI) tool was used to find out the risk of bias in the study.

Statistical Analysis

Meta-analysis was performed for outcomes reported by at least two studies with comparable data. For continuous variables, pooled mean differences (MDs) with 95% confidence intervals (CIs) were calculated. For dichotomous variables, pooled odds ratios (ORs) with 95% CIs were calculated. Statistical heterogeneity across studies was assessed using the I^2^ statistic and was reported for each pooled analysis. Forest plots were used to present the pooled effect estimates and corresponding 95% CIs. A fixed-effect model was applied when statistical heterogeneity was low, whereas a random-effects model was used when heterogeneity was moderate to high. A p-value of less than 0.05 was regarded as statistically significant. For interpretative clarity, lower I^2^ values were considered to indicate less between-study inconsistency, whereas higher I^2^ values were considered to indicate substantial heterogeneity.

Results

Study Selection

The study identified 18 articles based on the defined inclusion criteria. The articles were selected by separate reviewers, and those related to the title were included in this review. Full-text articles were searched for and selected according to the criteria outlined in the methodology. Following a comprehensive analysis of all the articles, 18 were chosen for review. The articles contained data regarding gender, study design, BMI, follow-up, major findings, MCs, counts for each Modic type, levels of changes, and associated disorders. Most of the studies compared in this review had the required details.

Study Characteristics

The overall number of patients in the 18 studies was 4,637. The patients' mean age and mean BMI were taken into consideration to compare with the age associated with MCs. Studies were based on prospective, retrospective, case-control, randomized control, cohort, and cross-sectional designs. Among these, eight were retrospective, three were prospective, two were case-control designs, two were prospective cohort studies, one was a cross-sectional cohort study, one was a longitudinal cohort study, and one was a randomized study design. According to the JBI grades of recommendation, 14 studies were classified as grade A, whereas four were classified as grade B. According to the level of evidence, two studies were level 1, four were level 2, and 12 were level 3 (Table 1).

**Table 1:** Summary of the findings and level of evidence NR: not reported; MCs: Modic changes; IVD: intervertebral disc

S. no.	Lead author (year)	Gender (male)	Gender (female)	Overall total no. of patients	No. of MCs	Overall findings	Study design	Follow-up	Key findings	Level of evidence
1	Hanne B. Albert and Manniche C 2007 [8]	NR	NR	166	81	Low back pain	Longitudinal cohort	14 months	MCs were strongly associated with low back pain.	II
2	Lavanya Dharmalingam and Kadirvelu S 2025 [9]	52	56	108	97	Lumbar degenerative disease	Cross-sectional longitudinal study	NR	The L4-L5 and L5-S1 levels are where MCs mostly occur. Type 2 was the most prevalent, and the occurrences of types 1 and 3 were roughly the same number of times.	III
3	Juhani H. Määttä et al. 2016 [10]	425	717	1142	282	Low back pain	Cross-sectional cohort study	NR	MC showed an independent correlation with continual severe low back pain and back-related problems.	II
4	Jietao Xu et al. 2019 [11]	172	104	276	94	Spinal stenosis, lumbar disc herniation	Retrospective study	29.6 months	Percutaneous endoscopic lumbar discectomy via a transforaminal approach reduced low back pain and radiculopathy in individuals with lumbar disc herniation.	III
5	Xiaoping Mu et al. 2020 [12]	167	154	321	138	Low back pain	Retrospective study	NR	A reduced intervertebral space was the sole component contributing to the formation of MCs.	III
6	Matilde Bianchi et al. 2015 [13]	87	139	226	141	Low back pain, lumbar facet injection	Retrospective study	Two to three years	The presence or absence of MCs does not affect the effectiveness of therapeutic lumbar facet joint injections.	III
7	Nam-Su Chung et al. 2021 [14]	32	54	86	72	Spondylolisthesis, degenerative disc disease, spinal stenosis	Retrospective case-control study	28.6 ± 12.1 (months)	Oblique lateral interbody fusion is not affected by vertebral endplate lesions	III
8	Mladen Djurasovic et al. 2012 [15]	28	23	51	31	Spondylolisthesis, spinal stenosis, scoliosis, tumour, infection	Retrospective study	24 months	Degenerative disc disease is effectively treated with lumbar fusion.	III
9	Kumarasamy D et al. 2022 [16]	202	107	309	86	Disc herniation	Prospective comparative cohort study	12 months	Modic alterations were associated with more severe disc degradation and higher total endplate scores.	I
10	Mark Hancock et al. 2011 [17]	16	14	30	3	Disc degeneration, disc herniation	Case-control design	NR	Disc degeneration and herniation, Modic alterations are more prevalent in acute low back pain.	III
11	Andreas Sørlie et al. 2012 [18]	112	66	178	129	Lumbar disc herniation, unilateral microdiscectomy	Prospective cohort study	12 months	Preoperative type 1 MCs show less substantial reduction in back pain following microdiscectomy.	II
12	Young-Min Kwon et al. 2009 [19]	119	232	351	92	Lumbar disc disease, posterior lumbar interbody fusion (PLIF)	Retrospective study	59.8 months	The presence of MC types 1 and 2 signals seems to be an indicator for posterior fusion. On the other hand, MC type 3 doesn't seem to be a good reason for posterior fusion.	III
13	Jiaxun Jiao et al. 2021 [20]	40	49	89	51	Disc herniation, lumbar spinal stenosis, lumbar spondylolisthesis	Retrospective study	24 months	MCs did not affect fusion rates or clinical outcomes.	III
14	Christian Hellum et al. 2012 [21]	73	81	154	131	Disc degeneration and low back pain in either one or both of the lower lumbar levels.	Randomized study	24 months	People with MC types 1 or 2 must be considered for surgical intervention.	I
15	Masatoshi Teraguchi et al. 2022 [22]	246	568	814	516	Disc degeneration, disc herniation	Prospective study	NR	MC types 1 and 2 are independently linked to pain characteristics.	II
16	Alessandra Splendiani et al. 2019 [23]	20	18	38	38	Non-specific low back pain	Prospective study	NR	Modic type 1 changes and pain intensity increase in the upright position.	II
17	F.M. Kovacs et al. 2012 [24]	143	161	304	249	Severe disk degeneration	Case control	NR	The presence of vertebral endplate changes in the lumbar spine was not associated with chronic low back pain.	III
18	I. Braithwaite et al. 1998 [25]	31	27	58	27	Discogenic low back pain	Retrospective study	NR	MCs seem to be a very precise sign of a painful IVD, according to discography.	III

Risk of Bias

**Figure 2:**
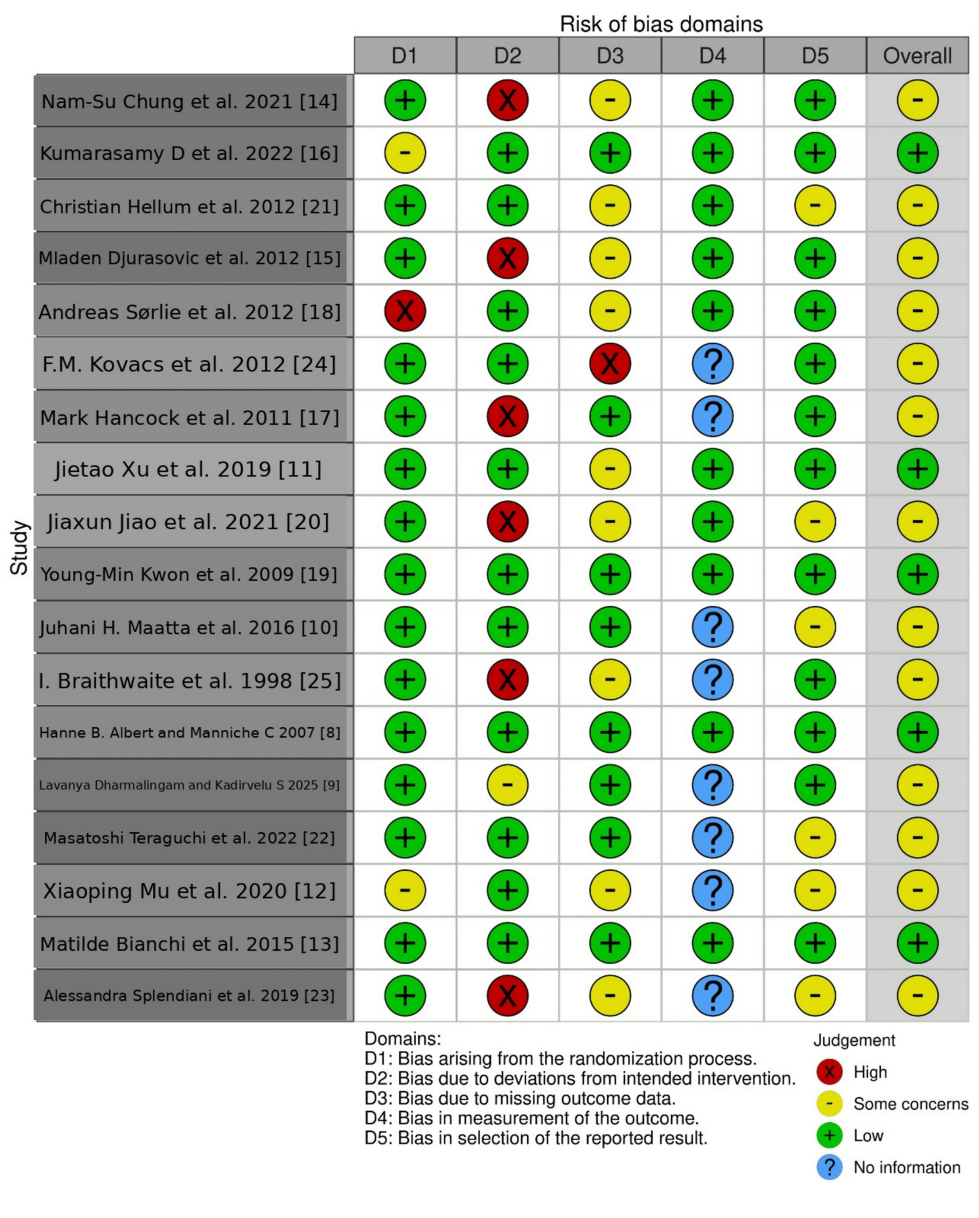
Risk of bias (traffic light plot) Studies included [8-25]

**Figure 3:**
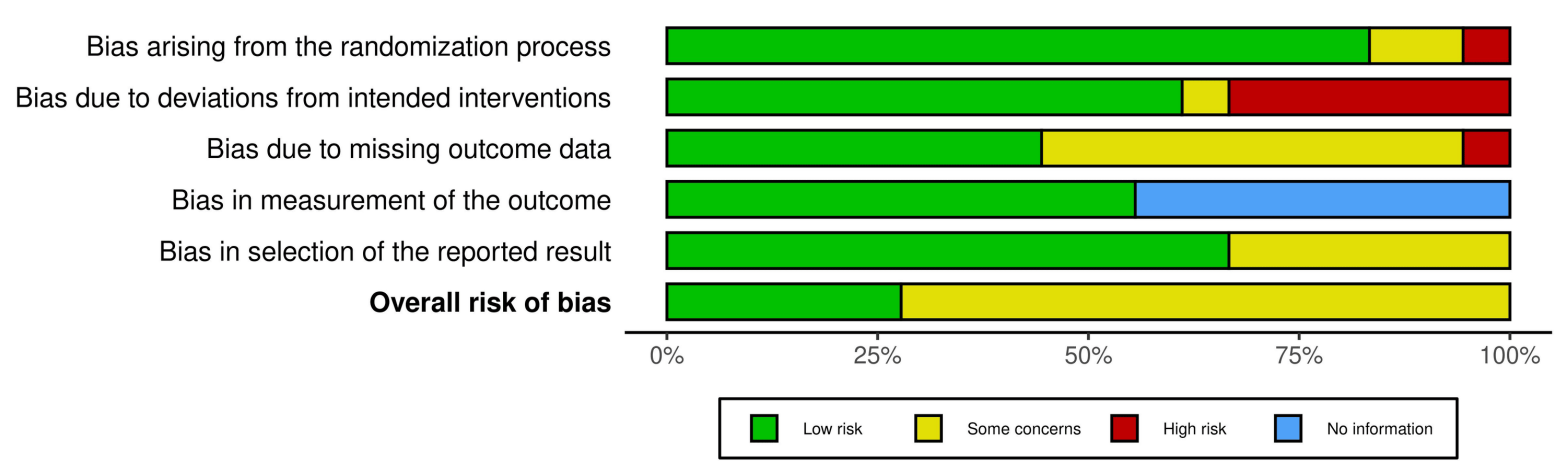
Risk of bias (summary plot)

Four studies were found to have low bias. The external variables that could affect the study's outcome were not identified in these studies. Other studies had external variables like smoking, which were found to be inconsequential, but can alter the desired outcome, and were found to be a risk factor. Risk of bias was measured by the Robvis visualization tool. Figures 2-3 show the risk of bias in studies using the traffic light plot and summary plot.

Results of individual studies

Age and BMI

In the 18 studies that were included for analysis, 11 studies included the mean age of the participants (Table 2). Among the studies, the MCs were found to be higher when the mean age was >40. Despite the fact that the cross-sectional cohort study had a larger sample size and a mean age of 52.9, MCs were determined to be less than 25% [10]. The other studies showed a direct relationship between age and vertebral endplate changes. The age of the participants with a BMI between 25 and 29 is always a precipitating factor for the Modic endplate changes. Age and BMI are considered risk factors for MCs.

**Table 2:** Overall mean age and patients with and without Modic changes SD: standard deviation; NR: not reported

S. no.	Lead author	Year	Overall mean age	SD	Modic changes		No Modic changes	
					Mean	SD	Mean	SD
1	Hanne B. Albert and Manniche C [8]	2007	-	-	NR	-	-	-
2	Lavanya Dharmalingam and Kadirvelu S [9]	2025	-	-	NR	-	-	-
3	Juhani H. Määttä et al. [10]	2016	53	-	53.9	6.3	52.6	6.5
4	Jietao Xu et al. [11]	2019	-	-	42.2	13.6	38.8	11.9
5	Xiaoping Mu et al. [12]	2020	-	-	52.64	11.69	48.99	13.66
6	Matilde Bianchi et al. [13]	2015	61.6	13.33	64.5532	13.1077	56.6	14.01
7	Nam-Su Chung et al. [14]	2021	64.7	9.1	-	-	-	-
8	Mladen Djurasovic et al. [15]	2012	47	-	-	-	-	-
9	Kumarasamy D et al. [16]	2022	-	-	43.1	10.3	42.2	13.4
10	Mark Hancock et al. [17]	2011	37	-	36.8	7.4	36.6	7.4
11	Andreas Sørlie et al. [18]	2012	41.2	12.1	42.5	10.7	40.9	12.5
12	Young-Min Kwon et al. [19]	2009	47.4	11.4	48.34	11.7806	46.9	12.75
13	Jiaxun Jiao et al. [20]	2021	-	-	57.51	9.2273	55.6	8.6
14	Christian Hellum et al. [21]	2012	40.9	7.1	-	-	-	-
15	Masatoshi Teraguchi et al. [22]	2022	63.6	13.1	-	-	-	-
16	Alessandra Splendiani et al. [23]	2019	47.4	5.2	-	-	-	-
17	F. M. Kovacs et al. [24]	2012	-	-	43	-	45	
18	I. Braithwaite et al. [25]	1998	42	10.5	-	-	-	-

**Figure 4:**
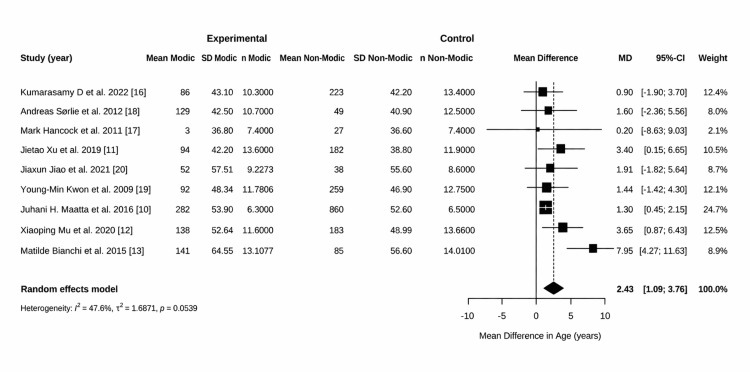
Forest plot of the mean difference (MD) in age between patients with and without Modic changes Studies included [16,18,17,11,20,19,10,12,13]

The forest plot (Figure 4) shows the MD in age between patients with MCs and those without. The MD is 2.43 years (95% CI: 1.09 to 3.76). Among the nine studies that were included, three showed a statistically significantly higher age for the Modic group [11-13], while the others showed a positive trend that did not reach individual significance. The study by Määttä et al. carried the greatest weight (24.7%) in the meta-analysis due to its large sample size (n = 1,142), which significantly increased the precision of the final estimate. There is moderate heterogeneity in the results (I^2^ = 47.6%) [10]. This suggests that while most studies show a positive trend, the magnitude of the age difference varies across different research populations. With respect to the BMI overall test for effect, a p-value of 0.705 was obtained, which is not statistically significant. The pooled analysis for BMI did not show a statistically significant association with MCs.

Gender

According to most authors, gender had a minimal role in MCs. Also, one thing to note is that gender can also not be ruled out entirely. In most research articles that collected participants' gender, the female population was marginally greater than the male population. Apart from the two studies that didn’t include gender data, nine had more female participants than male participants. A total of 2387 participants were female, which accounts for more than 50% of the study population. Although gender is not a major risk factor, a study by Wáng YX et al. found that there was a higher prevalence of LBP in females compared to males after the onset of menopause [26]. Gender is a risk factor with advancing age and high BMI.

**Figure 5:**
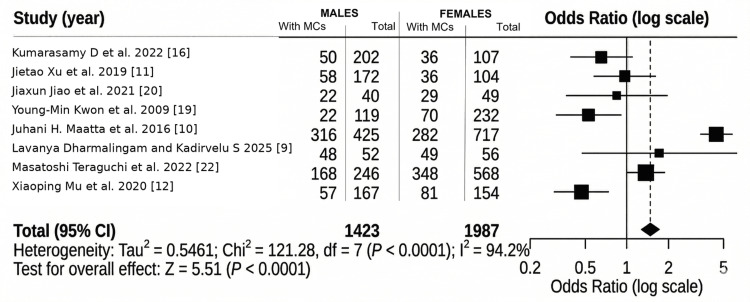
Forest plot showing Modic changes (MCs) in males and females Studies included [16,11,20,19,10,9,22,12]

The forest plot (Figure 5) evaluates whether males are more likely to present with MCs than females. The pooled analysis for gender showed that males had higher odds of presenting with MCs than females (OR: 1.51, 95% CI: 1.22 to 1.88). However, the heterogeneity for this analysis was substantial (I^2^ = 94.2%), indicating marked variation across studies. Therefore, this association should be interpreted with caution.

Spondylolisthesis

**Figure 6:**
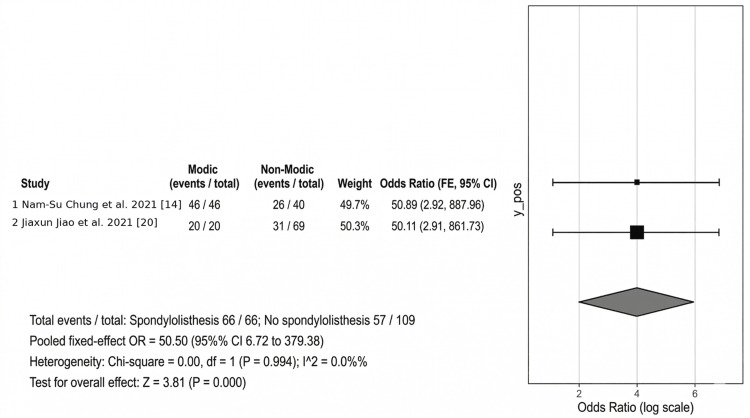
Spondylolisthesis as a risk factor for Modic changes Studies included [14,20]

In the three research articles that included spondylolisthesis, spinal stenosis was identified as a related condition. In the study by Chung et al., MCs were found to be higher, with 83.7% of the patients with spondylolisthesis indicating it as a risk factor for disc degeneration [14]. There is weak evidence to show a direct correlation between spondylolisthesis and MCs. Spondylolisthesis can be a novel cause for MCs. Djurasovic et al. demonstrated narrowed disc space is an indicator for lumbar fusion for clinical improvement [15]. In the pooled analysis of studies reporting spondylolisthesis, the summary estimate showed a strong association with MCs (pooled OR: 50.50, 95% CI: 6.72 to 379.38). However, this finding was derived from a limited number of studies and should be interpreted cautiously in view of the sparse evidence base (Figure 6).

Disc Herniation

Kumarasamy et al. stated that MCs were linked to greater total endplate scores and disc degeneration grades [16]. However, there was no discernible difference in clinical results between the various MC types. L4-L5 (49.5%) and L5-S1 (46.9%) were the most commonly reported levels of disc herniation. In terms of herniation type, 255 patients had extrusion; 80 had MCs; 42 had protrusion; three had MCs; and only 12 had sequestration, with Modic alterations in three of them.

Hancock et al. studied a controlled population with 30 patients who matched their inclusion criteria (herniation, annular tear, MCs) [17]. A total of 57% exhibited herniation, 48% had annular tears, and 10% showed Modic type 1 changes. The study was limited by the very small population, which was a risk factor for bias. Although MCs were reported, the changes do not substantiate the sample size in this study. In this study, herniation cannot be considered the sole cause of MCs.

According to Xu et al., among 94 individuals with MCs, the majority presented with disc herniation as the primary clinical condition. The data strongly indicate that herniation is a risk factor for Modic alterations [11]. The study concluded that patients who had MCs, particularly those with Modic type 1 changes, demonstrated a trend of reduced postoperative LBP.

Microdiscectomy

Sørlie et al. discussed the outcomes after unilateral microdiscectomy. The cohort study included 178 patients who underwent lumbar microdiscectomy. This research indicated that smoking was the only independent risk factor for limited improvement in back pain, and there appears to be a relationship between smoking and Modic type 1 changes [18]. The improvement in back pain was 26.9 (mean, SD 25.8) for non-smokers and 8.1 (mean, SD 28.6) for smokers (p = 0.009). There was no statistically significant improvement in back pain among smokers with MC type 1. Smoking was a risk factor for postoperative improvement in MC.

Posterior Lumbar Interbody Fusion (PLIF)

Kwon et al. discussed the outcome of PLIF with respect to different Modic types. For patients without vertebral end plate degeneration, the fusion rate was 96.5%. For those with MCs type 1, it was 80.8%; for those with MCs type 2, it was 83.6%; and for those with MCs type 3, it was 54.5%. Patients with MC type 3 had a significantly lower fusion rate than those with other types (p < 0.05). Patients with MC type 3 had considerably poor clinical outcomes and bony fusion rates. Lesions of MC type 3 do not seem to be a reliable indicator for posterior fusion [19].

Transforaminal Lumbar Interbody Fusion (TLIF)

Jiao et al. discussed the outcome of TLIF in their study. Of the 89 patients who underwent TLIF, 38 were in the MCs-0 group, 20 in the MCs type 1 group, and 31 in the MCs type 2 group. Every individual in this trial reported considerable improvement in their back and leg discomfort. MCs had no effect on clinical results or fusion rates. The study concluded regarding better results in terms of low back discomfort, and it raised the likelihood of cage subsidence [20].

LBP and Lumbar IVD​​​​​​ Degeneration

Hellum et al. analyzed 154 patients with chronic LBP and IVD degeneration. MCs type 1 was found in 26, MCs type 2 in 33, and both MCs type 1 and MCs type 2 in 15 of the cases. High Fear Avoidance Beliefs Questionnaire - Work (FABQ-W) was found to be a sign of future impairment in patients with LBP [21]. In another study by Määttä et al., a cross-sectional cohort study was conducted in a population of 1142, and 282 individuals were found to have MCs. LBP was classified into three categories based on the visual analog scale (VAS): VAS <3, VAS 3-5.9, and VAS >6. Määttä et al. found that individuals with MCs were older and had disc displacements more often (P < 0.001). This large-scale study with a larger sample size found that long-term severe LBP and back-related impairment were independently linked to MCs, and certain MC phenotypes and patterns were more strongly correlated than others [10].

In the retrospective studies taken up for analysis, out of 728 patients, 360 exhibited MCs. Bianchi et al. investigated the relationship between MCs and improvement after injection, using imaging-guided lumbar facet injections [13]. There were 83 MCs type 1 (36.72%), 58 MCs type 2 (25.66%), and 85 (37.61%) who had no MCs. The prediction of outcome in patients with MC type 1 was compared to those with MC type 2 at a span of one day, one week, and one month. While patients with MCs tend to exhibit better outcomes compared to those without, this disparity lacked statistical significance. In another retrospective study by Albert and Manniche, 12 of 81 patients with MCs underwent surgery for disc herniation, and nine exhibited MCs at one-year follow-up. The author considered IV disc herniation as a considerable risk factor for Modic alterations, even following surgery [8].

Mu et al. investigated the connections between lumbar sagittal parameters and MCs development using the Surgimap surgical planning software. Among all the studies analysed, this study discussed the higher incidence of female MCs (58.70%) than males (41.30%). Out of 138 patients, Modic type 2 was more common than the other Modic types. The logistic regression analysis indicated that diminished intervertebral space was a significant risk factor for the emergence of MCs at the L3/L4 level [12].

Level of Changes

**Figure 7:**
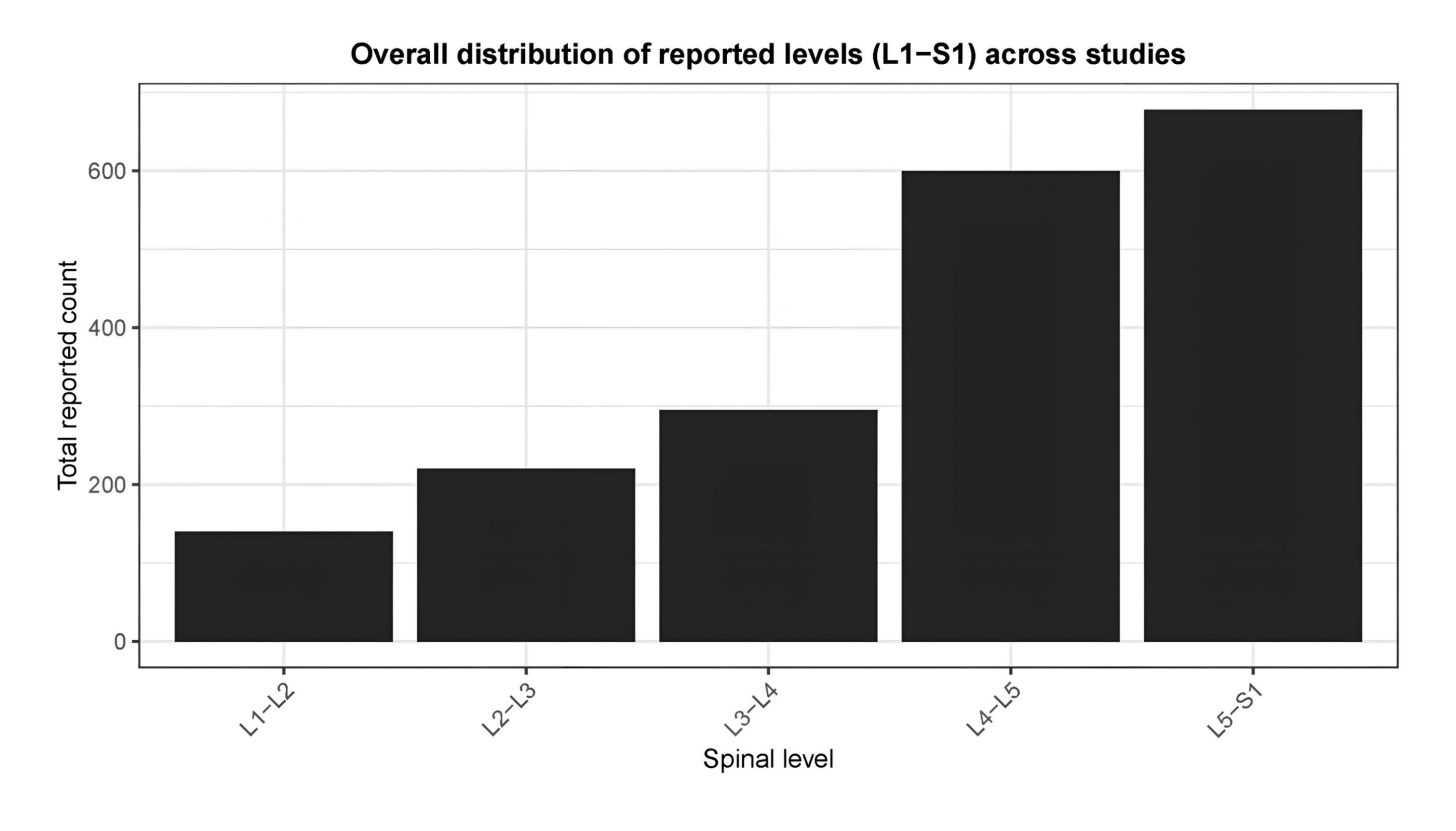
The distribution of Modic changes in the lumbar IV discs (L1-S1)

Ten studies reported the level of MCs. L4-L5 and L5-S1 were the most common sites of Modic endplate changes and were considered a strong risk factor for the onset of MCs. L1-L2 was reported in one study, and L2-L3 in two studies. L3-L4 was the next common level. Dharmalingam and Kadirvelu carried out a cross-sectional longitudinal study to gather data on chronic LBP resulting from lumbar IVD degenerative disease. Among 108 patients, 10 instances of type 1, 76 instances of type 2, and 11 instances of type 3 MCs were found. The study meticulously examined the correlation between the lumbar IVD level and the endplate exhibiting MCs. The study indicated that MCs occur more commonly at the end plates of the lower lumbar vertebrae, specifically at the L4-L5 and L5-S1 levels. Type 2 MCs were the most common, whereas types 1 and 3 exhibited approximately equal occurrences. The majority of the reported counts are concentrated at the L4-L5 and L5-S1 levels (Figure 7) [9]. Teraguchi et al. reported high counts across all levels, including L1-L2. The study has a significantly higher scale (over 300 reports) compared to the others (Table 3) [22]. Also, the overall distribution of the MCs in the studies employed in this review is shown in Table 4.

**Table 3:** Summary of studies showing the most affected IVD with MCs IVD: intervertebral disc; MCs: Modic changes

Study	Most affected IVD level	Observed distribution and pattern of MCs
Jietao Xu et al. 2019 [11]	L4-L5	Peak reporting at L4-L5, followed by L5-S1.
Xiaoping Mu et al. 2020 [12]	L5-S1	Focused exclusively on the L4-L5 and L5-S1 segments.
Matilde Bianchi et al. 2015 [13]	L5-S1	Reports start at L3-L4 and increase significantly toward L5-S1.
Nam-Su Chung et al. 2021 [14]	L4-L5	High overall numbers peaking at L4-L5, with notable L3-L4 and L5-S1 reports.
Kumarasamy D et al. 2022 [16]	L4-L5 and L5-S1	High and roughly equal reporting for both lower lumbar segments.
Andreas Sorlie et al. 2012 [18]	L5-S1	Significant changes from L3-L4 to L5-S1, peaking at the lumbosacral junction.
Jiaxun Jiao et al. 2021 [20]	L4-L5	A clear peak at L4-L5, with lower counts at L3-L4 and L5-S1.
Christian Hellum et al. 2012 [21]	L5-S1	Primarily reports count for L4-L5 and L5-S1; zero reports for L1-L3.
Masatoshi Teraguchi et al. 2022 [22]	L5-S1	Exceptionally high numbers (>300) across all levels, including upper lumbar (L1-L3).
I. Braithwaite et al. 1998 [25]	L5-S1	Shows a gradual increase in reports starting at L3-L4 and peaking at L5-S1.

**Table 4:** Overall distribution of the MCs types NR: not reported; MCs: Modic changes

S. no.	Lead author	Year	Overall total no. of patients (N)	No. of patients with MCs	No. of patients without MCs	MCs type 1	MCs type 2	MCs type 3	L1-L2	L2-L3	L3-L4	L4-L5	L5-S1
1	Hanne B. Albert and Manniche C [8]	2007	166	81	85	48	22	2	-	-	-	-	-
2	Lavanya Dharmalingam and Kadirvelu S [9]	2025	108	97	11	10	76	11	-	-	-	-	-
3	Juhani H. Määttä et al. [10]	2016	1142	282	860	81	201	NR	-	-	-	-	-
4	Jietao Xu et al. [11]	2019	276	94	182	44	50	NR	-	-	7	52	35
5	Xiaoping Mu et al. [12]	2020	321	138	183	42	96	NR	-	-	-	45	74
6	Matilde Bianchi et al. [13]	2015	226	141	85	83	58	NR	-	-	4	13	21
7	Nam-Su Chung et al. [14]	2021	86	72	14	33	22	17	-	4	28	64	29
8	Mladen Djurasovic et al. [15]	2012	51	31	20	10	18	3	-	-	-	-	-
9	Kumarasamy D et al. [16]	2022	309	86	223	18	68	NR	-	-	-	43	40
10	Mark Hancock et al. [17]	2011	30	3	27	3	NR	NR	-	-	-	-	-
11	Andreas Sørlie et al. [18]	2012	178	129	49	29	100	NR	-	-	14	47	68
12	Young-Min Kwon et al. [19]	2009	351	92	259	26	55	11	-	-	-	-	-
13	Jiaxun Jiao et al. [20]	2022	89	51	38	20	31	NR	-	-	6	28	17
14	Christian Hellum et al. [21]	2012	154	131	23	26/61	33/54	NR	-	-	-	19	45
15	Masatoshi Teraguchi et al. [22]	2022	814	516	298	63	326	22	140	216	233	280	334
16	Alessandra Splendiani et al. [23]	2019	38	38	0	38	NR	NR	-	-	-	-	-
17	F. M. Kovacs et al. [24]	2012	304	249	55	10	120	1	-	-	-	-	-
18	I. Braithwaite et al. [25]	1998	58	27	31	6	21	NR	-	-	3	9	15

Discussion

**Figure 8:**
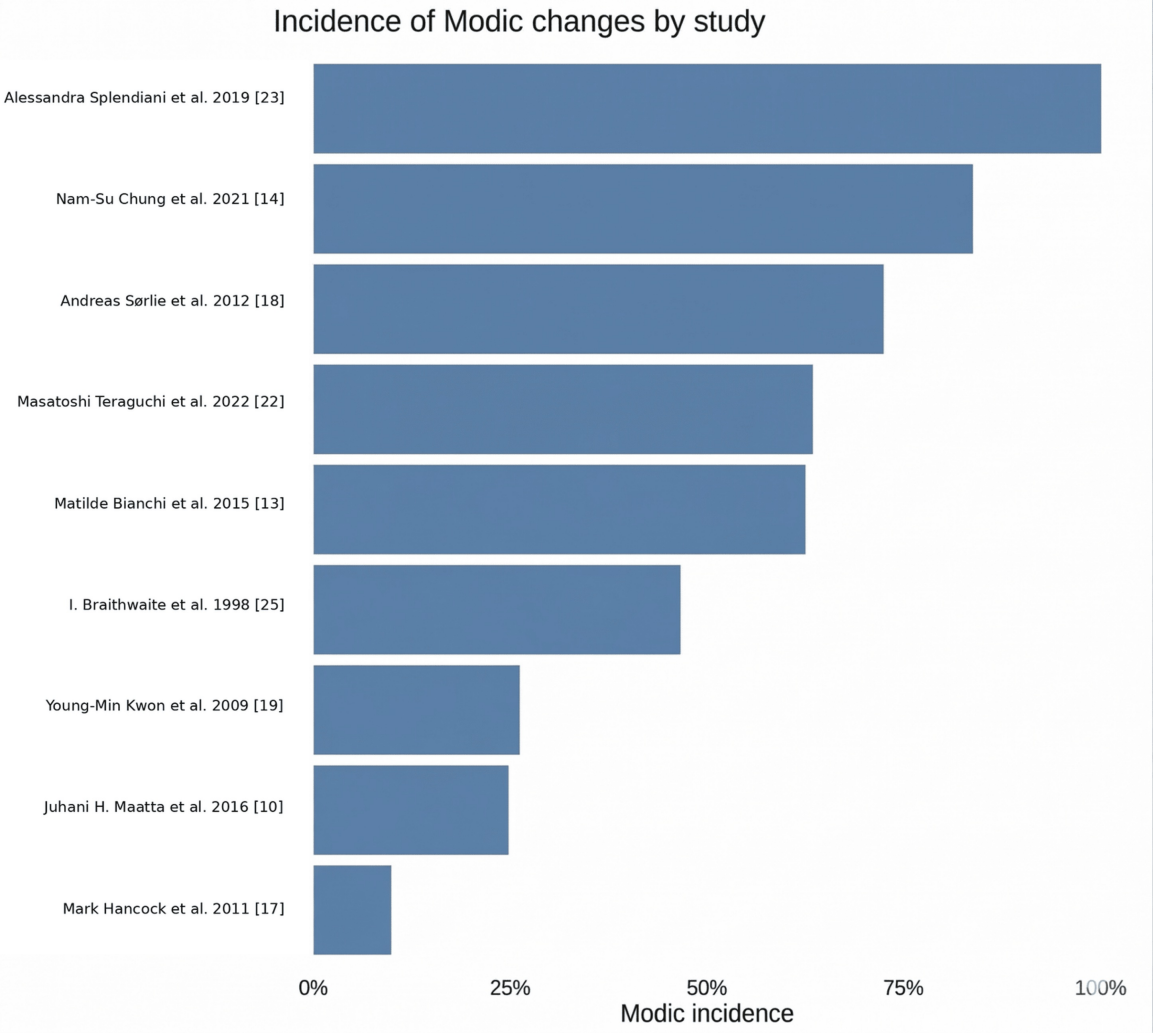
The incidence of MCs among different studies MCs: Modic changes Studies included [23,14,18,22,13,25,19,10,17]

The discussion examines the relationship among demographic factors, spinal pathologies, and clinical outcomes in patients exhibiting MCs. The comparison of the mean age difference (from the forest plot) (Figure 4) and the incidence rates (from the bar chart) (Figure 8) revealed patterns that indicate the relationship between aging and the emergence of MCs.

How Age Affects MCs

The two sets of data together show that there is a link between exposure and response: groups with a greater average age are likely to have a much higher rate of MCs.

High incidence/high age: Researchers like Splendiani et al. (indicated an incidence of almost 100%) [23] and Bianchi et al. analyzed older, symptomatic populations. Bianchi et al.'s study had a huge age difference in the forest plot, which is why the incidence rates are quite high (Figure 4) [13].

Low incidence/lower age: Research by Määttä et al. and Hancock et al. suggested both a low incidence (below 30%) and a smaller age difference. These studies frequently examine younger cohorts or the general community instead of specific spinal patients [10,17].

Clinical Significance Versus Statistical Significance

The forest plot demonstrates that there is a statistically significant age difference of 2.43 years. However, when compared to the incidence chart, it seems that biological age is more important than chronological age. The modest mean age difference indicates that MCs are not solely attributable to aging; rather, they are likely induced by specific events, such as disc herniation or mechanical stress, which occur more frequently as one ages. The wide variation of incidence among the studies shows that how patients are chosen is a better predictor of Modic alterations than just age.

Progressive Nature of Changes

Type 1 (inflammatory): Often seen in the initial stage of the Modic spectrum, representing acute stress.

Type 2 (fatty): Dominates the older cohorts and high-incidence studies, representing a chronic state of degeneration.

Demographic Interpretation of the Age, Gender, and BMI

The meta-analysis confirms a statistically significant link between advancing age and the incidence of MCs, exhibiting an MD of 2.43 years between the MC and non-MC groups. Määttä et al.'s study (2016) found that the prevalence of MCs stayed below 25% in a group of people with an average age of 52.9. However, the evidence on a larger scale indicated that 40 years serves as a threshold for higher incidence [10]. This shows that MC is caused by mechanical stress and the natural aging of the vertebral endplate.

The findings related to gender exhibit a striking disparity. Gender has a small effect, but the forest plot shows a substantial OR of 1.51, which means that males are more likely to be affected (Figure 5). This factor indicates the occupational susceptibility to endplate alterations in males. In the study by Wáng et al., males showed more radiographic change, but females experienced higher pain prevalence post-menopause [26]. This indicates that hormonal fluctuations may intensify the clinical manifestation of MC, despite structural alterations being more common in men.

**Figure 9:**
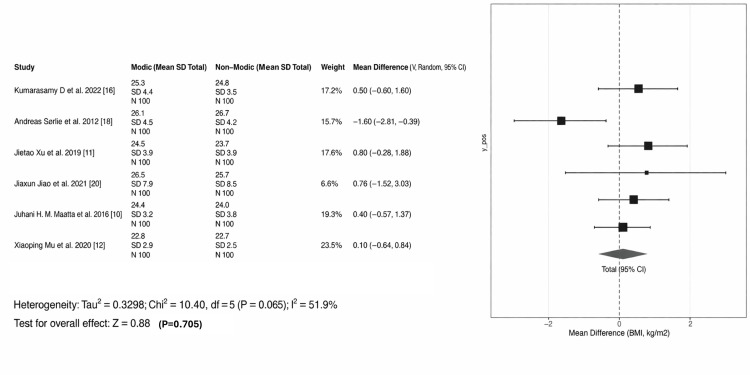
Forest plot of BMI and MCs BMI: body mass index; MCs: Modic changes Studies included [16,18,11,20,10,12]

The overall meta-analysis for BMI indicates a non-significant p-value of 0.705 (Figure 9). Although a BMI between 25 and 29 may act as a precipitating factor in specific cohorts, it is not a universal predictor of MCs. The onset of MCs depends more on the distribution of load and the integrity of the endplate rather than body mass alone.

Anatomical Distribution

**Figure 10:**
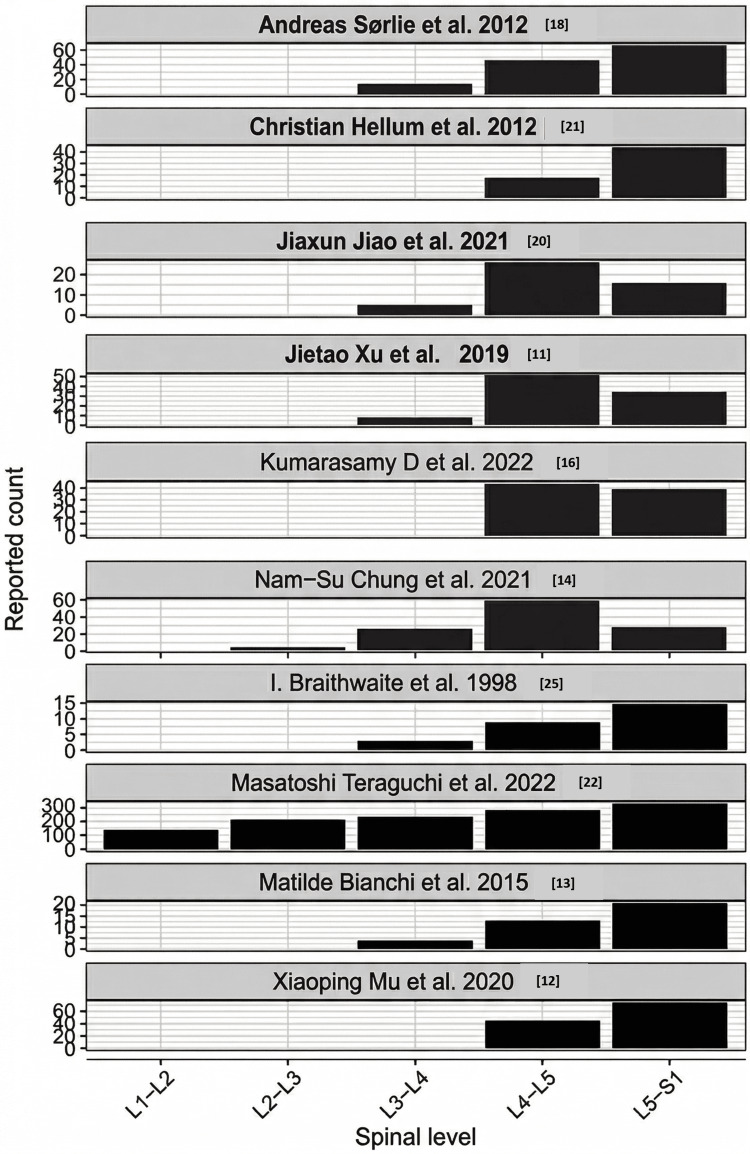
Studies showing the concentration of MCs at different IVD levels MCs: Modic changes; IVD: intervertebral disc Studies included [18,21,20,11,16,14,25,22,13,12]

Among the 10 studies where the levels were reported, the concentration of MC was found to be higher at the L4/L5 and L5/S1 levels (Figure 10). This suggests the axial weight-bearing areas and the shear forces were highest at the endplates of these levels. Dharmalingam and Kadirvelu reported a high prevalence of type 2 MCs at these levels [9]. This suggests a progression toward fatty marrow replacement in response to chronic degenerative stress.

Spondylolisthesis and Disc Herniation

The meta-analysis gives strong evidence that MC is not an isolated phenomenon but rather a component of a broader degenerative disorder.

Spondylolisthesis: The link between spondylolisthesis and MC is very strong, with an OR of 50.50. Repeated microtrauma at the endplates in spondylolisthesis hastens the progression of MC.

Disc herniation: The research conducted by Kumarasamy et al. and Xu et al. corroborates that herniation, especially extrusion, serves as a principal catalyst of MC [16,11]. The expansion of the nucleus pulposus into the vertebral body triggers the type 1 (inflammatory) MC signal.

The research by Määttä et al. demonstrated that Modic alterations represent a unique risk factor for severe and incapacitating LBP in middle-aged women [27].

Smoking and Type 1 MC

Sørlie et al. concluded that smokers with type 1 MC showed almost no improvement in back pain (8.1) compared to non-smokers (26.9). Smoking is known to impair microcirculation, which is vital for endplate nutrition [18].

Surgical Efficacy: PLIF Versus TLIF

Modic types 1 and 2: Although TLIF is less susceptible to MC, Jiao et al. identified a heightened risk of cage subsidence in their study [20]. The endplate has suffered damage; thus, it might not be strong enough to hold a synthetic cage under strain.

Modic type 3 (sclerotic): These patients have the most uncertain prognosis, with fusion rates in PLIF declining to 54.5%. The hardening of the bone decreases the blood flow needed for the bony bridge to form.

Määttä et al.'s study, which included a wide group of people, showed that MC is connected to long-term severe LBP on its own [10]. Nonetheless, the clinical response to conservative treatments, including facet injections, remains uncertain. Bianchi et al. indicated a propensity for enhanced outcomes in MC patients; nevertheless, the absence of statistical significance suggests that MC should not be the sole factor for deciding between injection and surgery [13].

Study Limitations

Subtype heterogeneity: The studies included showed a lot of differences in how they reported Modic subtypes. Consequently, determinations on the unique therapeutic effects of type 2 compared to type 3 modifications could not be validated.

Classification variance: The research employed diverse criteria to delineate and categorize MCs. This lack of uniformity made the data different from each other. Gao et al. took a new technique by using deep learning to find MCs [28]. This deep learning method helps find the MCs, but it has its limits.

## Conclusions

MCs are a visual map of the spine's history. The changes reveal that there have been herniations, spondylolisthesis, and disc degeneration in the past. The data reviewed highlight that MCs are complex, influenced by age, male gender, and mechanical instability. Future clinical guidelines ought to emphasize preoperative MRI phenotyping, specifically differentiating between inflammatory type 1 and sclerotic type 3, to enhance patient expectation management and facilitate the selection of suitable fusion techniques.
